# A candidate nanoparticle vaccine comprised of multiple epitopes of the African swine fever virus elicits a robust immune response

**DOI:** 10.1186/s12951-023-02210-9

**Published:** 2023-11-14

**Authors:** Jinxing Song, Mengxiang Wang, Lei Zhou, Panpan Tian, ZhuoYa Sun, Junru Sun, Xuannian Wang, Guoqing Zhuang, Dawei Jiang, Yanan Wu, Gaiping Zhang

**Affiliations:** 1 International Joint Research Center of National Animal Immunology, College of Veterinary Medicine, Henan Agricultural University, Zhengzhou, 450046 China; 2 Longhu Laboratory of Advanced Immunology, Zhengzhou, 450046 China; 3 Ministry of Education Key Laboratory for Animal Pathogens and Biosafety, College of Veterinary Medicine, Henan Agricultural University, Zhengzhou, 450046 China; 4 School of Advanced Agricultural Sciences, Peking University, Beijing, 100871 China

**Keywords:** African swine fever, Nanoparticles, XCL1, NanoFVax, Ferritin, Vaccine candidate

## Abstract

The African swine fever (ASF) pandemics pose a significant threat to the global swine industry, and the development of safe and effective vaccines is a daunting but necessary challenge. The level and persistence of immunity are very important for the effectiveness of the vaccine. Targeting antigens to antigen presenting cells (APCs) can greatly enhance immunogenicity. In this study, we developed a self-assembled nano-ASFV vaccine candidate (NanoFVax) targeting DCs, by covalently coupling the self-assembled 24-mer ferritin with the dominant B and T cell epitopes of the highly immunogenic ASFV antigen (p72, CD2v, pB602L and p30) and fused with the chemokine receptor XCL1 (a DC targeting molecule) through the SpyTag/SpyCatcher protein ligase system. Compared to monomeric protein, the nanoparticle vaccines can induce a more robust T-cell response, and the high-level antibody response against ASFV can last for more than 231 days. Therefore, the NanoFVax is a novel and promising vaccine candidate for ASFV.

**Graphical Abstract:**

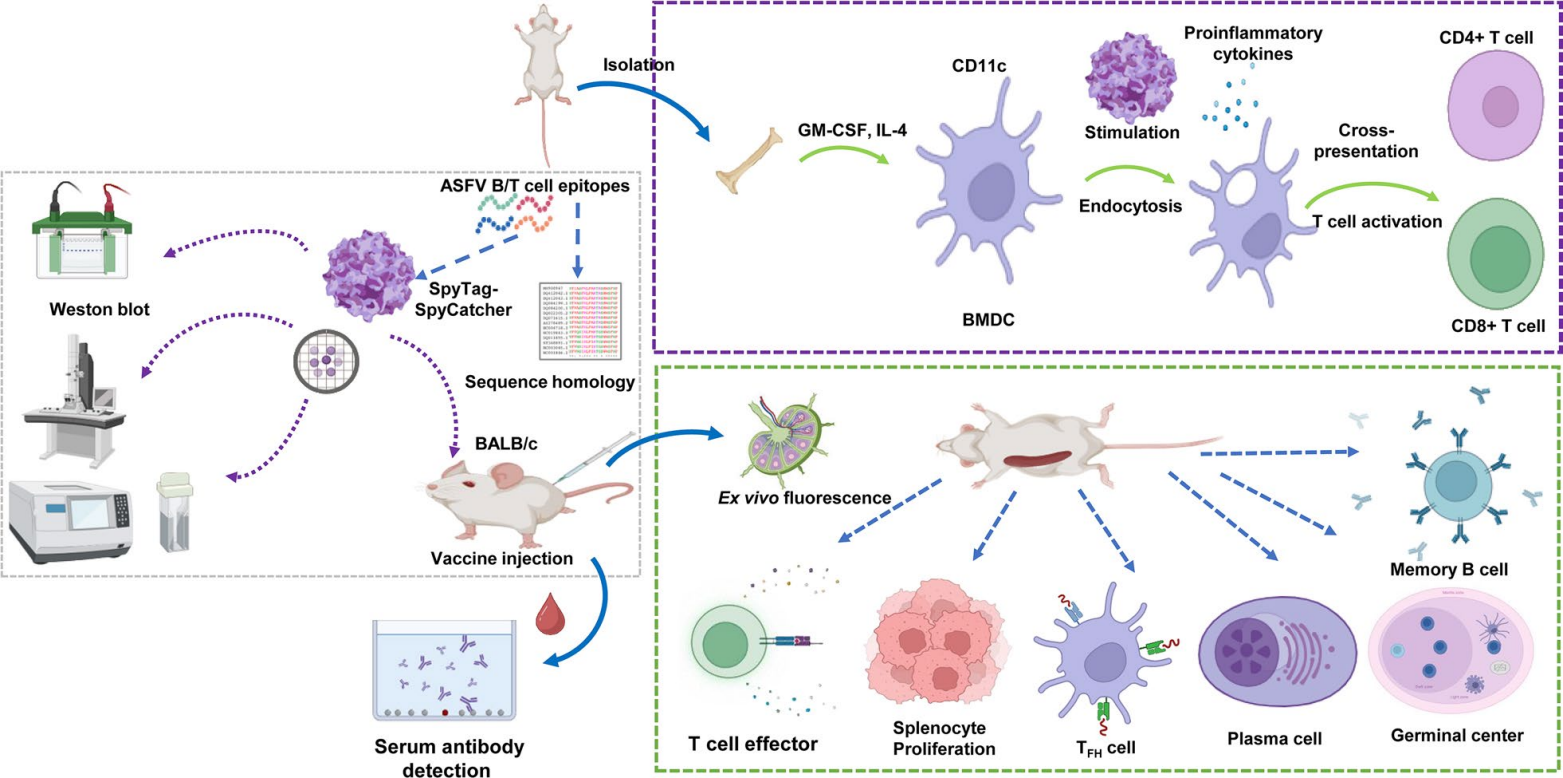

**Supplementary Information:**

The online version contains supplementary material available at 10.1186/s12951-023-02210-9.

## Introduction

African swine fever virus (ASFV) is a highly contagious pathogen with a fatality rate of almost one hundred percent in domestic and wild pigs. ASF is listed as a legally notifiable disease that must be reported to the World Organization for Animal Health (WOAH) [1]. There is no vaccine or other treatment strategy except for biosafety prevention and control at present [2]. Since the 2018 entry of ASFV into China, the pig industry has been heavily impacted, creating an urgent need for a safe and effective ASFV vaccine that can neutralize current ASFV viruses, with the potential for protection from future mutant strains. Such vaccines would be comprised of B-cell and T-cell epitopes that induce protective humoral and cellular immune responses [3]. The identification of target epitopes will be a key step in the development of ASF vaccines and diagnostic reagents.

Several factors have hindered the development of ASF vaccine, including the complexity of ASFV structure, the lack of knowledge of virus-host interaction, and the unclear understanding of protective immune mechanisms [4]. Recent studies have determined the multi-layer structural details and assembly of ASFV at atomic resolution, paving the way for vaccine research and development [5, 6].

Several ASFV proteins, including p72, p30 and CD2v, have been reported to be able to induce neutralizing antibodies in pigs [7]. Major capsid protein p72 is the main structural component of the ASFV, accounting for 31% ~ 33% of the total mass of ASFV, and is also one of the main target antigens [8]. The pB602L protein is a non-structural protein that serves as a molecular chaperone for p72. An absence of pB602L results in an abnormal “zipper-like” structure, unable to assemble normal icosahedral virus particles. Previous studies have shown that pB602L and p30 were highly antigenic and can also be used to develop ASF vaccines and diagnostic tools [5, 9]. CD2v is an important protective antigen of ASFV, providing serotype-specific cross-protective immunity [10]. Studies have shown that CD2v is the only known viral homologue of cellular CD2, a T cell protein involved in the co-regulation of cell activation, so that epitopes of CD2v are also necessary for the construction of ASF subunit vaccines [11].

Recently, nano-vaccines have received increasing attention due to their capacity to accumulate in lymph nodes, self-assemble, and present antigens [12, 13]. Nano-sized particles are easily taken up by cells, improving the efficacy of vaccines compared with traditional ones. Moreover, compared with small-molecular and soluble vaccines, nano-vaccines are easily recognized by antigen-presenting cells (APCs) [14]. Dendritic cells (DCs) play an important role in antigen presentation, and are considered to be the major professional APCs in vivo, playing a key role in the induction and regulation of antigen-specific T and B cells. DCs capture antigen, then process and present antigenic peptides to T cells via MHC class I and class II molecules [15]. Murine DCs that cross-present antigens express chemokine receptor XCL1 with XCL1 as the ligand [16]. So targeted XCL1 can increase the efficacy of vaccines [17].

Epitope vaccine is a new type of animal vaccine developed in recent years. Accurate identification of short and weakly antigenic B-cell epitopes and T-cell epitopes is a critical step in designing an effective vaccine. In addition, effective delivery for epitope vaccines is necessary. Self-assembled nanocarrier vaccines, such as ferritin, provide for a non-viral platform for caged proteins [18]. Ferritin can self-assemble into highly ordered 24 mer polymers, forming multiple surfaces for the display of antigenic epitope, greatly improves immunogenicity and vaccine efficacy with minimal heterogeneity [19]. Epitope-based nanoparticle vaccines have revolutionized the development of vaccines for a wide range of diseases [20]. Moreover, subunit vaccines expressed in prokaryotic systems are significantly less expensive than in eukaryotic expression systems.

Although no database of current ASFV epitopes exists, analysis of ASFV epitopes is ongoing with information becoming more abundant. Herein, we used bioinformatics tools to predict and validate B-cell and T-cell epitopes for evaluation of epitope conservation.

The aim of this study was to develop a nanoparticle vaccine targeting DCs, based on immune-dominant ASFV antigenic epitopes packaged using ferritin as a self-assembling carrier. This nanoparticle vaccine was shown to induce high and sustained levels of specific antibodies and antigen-specific T-cells, at low antigen dose. The high titer antibody levels induced by the nanoparticle vaccine could theoretically provide protection from ASFV infection. Moreover, the vaccine development approach provided valuable insights into ASFV vaccine production for next-generation ASFV vaccine candidates.

## Materials and methods

### Selection and identification of immunodominant B and T cell epitopes

The most antigenic regions of p72, CD2v, pB602L and p30 were predicted by ABCpred website (http://crdd.osdd.net/raghava/abcpred/) and the Protean program (DNAStar, Madison, USA) using the GenBank reference sequence (GenBank No. MK333180.1).

The reactivity of the predicted B-cell epitopes to anti-ASFV serum (purchased from China Veterinary Culture Collection Center) was validated by Dot-ELISA. Briefly, 2 μg of each synthesized epitope peptide was applied to a nitrocellulose membrane, blocked with 5% BSA for 1 h at 37 °C, washed three times with PBST, and then incubated with anti-ASFV serum for 1 h at 37 °C, followed by the addition of peroxidase-labeled mouse anti-porcine IgG. Proteins were visualized using enhanced chemiluminescence (ECL) chromogenic substrate (New Cell & Molecular Biotech, Suzhou, China) according to the manufacturer's protocol.

Potential T-cell epitopes were screened from CD2v and p72 using NetMHC 4.0 software (https://services.healthtech.dtu.dk/services/NetMHC-4.0/), which predicts peptides that bind to a large number of SLA class I molecules. We mapped nonapeptides that bound to SLA-I molecules, selected de novo peptides with high predicted binding affinity (consensus score < 0.5) and intermediate predicted binding affinity (consensus score = 0.5 ~ 1.0) to SLA-1*0401 allele. 8-week-old female BALB/c mice were injected intramuscularly with ASFV p72 and CD2v recombinant protein to validate immunodominant T-cell epitopes, and the recombinant protein was maintained in our laboratory and stored at −80 °C. The enzyme-linked immunospot (ELISpot) assays were performed using mouse IFN-γ ELISpot kits (Dakewe, Beijing, China) and carried out in accordance with the instruction manual.

The protein sequences of p72, CD2v, pB602L and p30 of different genotypes of ASFV isolates were compared with those of ASFV/HLJ/2018 isolate. Sequence conservation of the identified epitopes was performed using ESPript 3.0 (http://espript.ibcp.fr/ESPript/ESPript/).

Moreover, the structural proteins screened here are highly antigenic and expressed at early or late stages of ASFV infection. Therefore, the spatial characteristics of the epitopes were analyzed to improve the specificity of the vaccine by their localization in the three-dimensional (3D) model. Finally, predicted 3D models were established using Phyre2 (http://www.sbg.bio.ic.ac.uk/phyre2), while molecular characterization was presented using PyMol.

### Design of the nanoparticle fusion immunogen

Ferritin (GenBank No. WP_000949190.1) was subcloned into the pET-28a ( +) vector with SpyCatcher (GenBank No. MF974388.1) at the N-terminus, and the recombinant plasmid is abbreviated as SC-Ferritin. The SpyTag (GenBank No. MF974389.1) was located at the N-terminus and linked to the XCL1 receptor (GenBank No. NP_032536.1) through the GSGGSG linker, and the dominant epitopes in Tables 1 and 2 were sequentially ligated through the GGGS flexible linker and the tandem was repeated three times for each epitope, followed by the *BamHI/XhoI* restriction site, subcloned into the N-terminus of the pET-28a ( +) vector, in the following we refer to the recombinant plasmid abbreviated as ST-XME. SC-Ferritin and ST-XME were expressed as fusion proteins with a C-terminal 8 × His-tag.

**Table1:** Predicted B cell epitopes from ASFV pB602L, p30, p72 and CD2v proteins were determined with ABCpred and the protean prediction tool of the DNAStar software (Lasergene 12)

Protein	No	Sequence	Position (aa)
pB602L	1	DGKADKIIL	13–21
	2	SCKTQTQKSKE	124–134
	3	NVDTCASTCTS	189–199
	4	FKNDSRVAF	39–47
	5	TTKTLLSEL	87–95
	6	VDSSNQQKV	327–336
	7	TLKQETNDVPSES	101–113
	8	PPQDTFYKW	284–292
p30	9	LFEEETESSAS	90–100
	10	HEKNDNETN	105–113
	11	KTDLRSSSQV	16–25
	12	FEQEPSSEV	123–131
	13	PKDSKLYML	132–140
	14	TEHQAQEEWNMI	75–86
	15	IEQYGKAPD	148–156
	16	GTPLKEEEKE	172–181
p72	17	NSRISNIKNV	27–36
	18	TGTPTLGNKLTFGIP	81–95
	19	QTFPRNGYDWD	135–145
	20	HFPENSHNIQTA	279–290
CD2v	21	NDNNDINGVSWNF	34–46
	22	GKAGNFCECSN	58–68
	23	IFPHNDVFDTTYQ	84–96
	24	CKKNNGTNTNIYL	129–142

**Table 2:** Predicted T cell epitopes derived from ASFV CD2v and p72 for the SLA-1*04:01 allele using the IEDB analysis tool

Protein	No	Sequence	Position (aa)
CD2v	1	LVNEFPGLF	17–25
	2	YTNESILEY	150–158
	3	SALKWPIEY	37–45
	4	KLTFGIPQY	19–27
	5	LIDKFPSKF	59–67
p72	6	LLPKPYSRY	61–69
	7	YLTLSSNYF	119–127
	8	ISDISPVTY	522–530
	9	PSTQPLNPF	290–298
	10	NITYNCTNF	117–125

### Purification and identification of proteins

SC-Ferritin and ST-XME were expressed in *E. coli* BL21 (DE3) strain (TsingKe, Beijing, China) containing chaperone plasmid pTf16 chaperone plasmid (Takara, San Jose, CA, USA) to enhance protein folding. The protein production was induced for 12 h by addition of 0.5 mM isopropyl-β-d-thiogalactopyranoside (IPTG) and 1.5 mg mL^−1^ of arabinose at OD600 = 0.5. The proteins were purified by Ni–NTA column (GE Healthcare, UK), and the endotoxin levels were evaluated by the Limulus Amebocyte Lysate (LAL) assay kit (Solarbio, Beijing, China). The purified protein was verified by SDS-PAGE. For ST-XME, the proteins are in monomeric form, which we call monomer. Purified monomer was incubated overnight at 4 °C with SC-Ferritin at a 24:1 molar ratio. The monomer and SC-Ferritin-conjugated nanoparticles, which we referred to as NanoFVax. Nanoparticles size diameter was characterized by dynamic light scattering (DLS) (Wyatt Technology, Goleta, CA, USA). Models of NanoFVax were generated using AlphaFold2.

### Morphological detection of proteins

The morphology of the nanoparticles was observed by negative staining transmission electron microscopy (TEM). Briefly, purified samples were diluted in PBS to a final concentration of 0.1 mg mL^−1^. Subsequently, 5 μL of the sample suspension was dropped onto copper mesh with film and allowed to stand for several minutes, then the excess liquid was removed with filter paper, uranyl acetate solution was dripped for 1 min, and then dried for electron microscopic observation. Images were taken with a Tecnai G2 spirit BioTwin (FEI) at Henan Agricultural University.

### In vitro bone marrow derived dendritic cell (BMDC) stimulation

BMDCs were differentiated from bone marrow cells isolated from BALB/c mice and cultured in 10% FBS (Gibco, Grand Island, NY, USA) RPMI-1640 medium containing 1% penicillin/streptomycin (Solarbio, Beijing, China) supplemented with IL-4 (10 ng mL^−1^, R&D Systems) and 20 ng mL^−1^ GM-CSF (20 ng mL^−1^, R&D Systems, BiosPacific, Emeryville, CA, USA) at 37 °C and 5% CO_2_. Cells were harvested on days 7 and used immediately. BMDCs isolation results in a yield of approximately 5–7 × 10^6^ total cells/mouse. The immature BMDC (1 × 10^5^) were plated in 96-well plates and then treated with lipopolysaccharide (LPS) (100 ng mL^−1^, Solarbio, Beijing, China) for 48 h. The polarized Th1 and Th2 responses were investigated by determining the secretion of various cytokines. Culture supernatants were harvested for detection of cytokine (IL-2 and IL-10) levels using mouse IL-2 ELISA kit (Invitrogen, Waltham, MA, USA) or mouse IL-10 ELISA Kit (Ruixin Biotech, Nanjng, China) according to the manufacturer's protocol. Cells were detached from culture dishes using 0.25% (v/v) trypsin–EDTA, resuspended in PBS buffer, and DC maturation markers were assessed by flow cytometry. Cell surface costimulatory molecules were detected using FITC-labeled anti-mouse CD80 + , anti-mouse CD86, and PE-labeled anti-mouse MHC-II. All antibodies were purchased from BioLegend (San Diego, CA).

### Cellular uptake and intracellular distribution of NanoFVax

To explore the cellular uptake of nanoparticles, NHS-FITC-tagged nanoparticles were assessed by confocal fluorescence imaging. DC2.4 cells were cultured at low density in 24-well plates overnight. NanoFVax was labeled using a FITC conjugation kit (MCE, HY-66019) based on the manufacturer’s instructions. FITC-labeled NanoFVax (20 μg well^−1^) was added for 24 h. Supernatants were gently discarded, cells were washed, fixed with 4% paraformaldehyde (Solarbio, Beijing, China) for 30 min at room temperature, rinsed with 0.1% PBS, and permeabilized with 0.1% Triton X-100/PBS for 10 min. Phalloidin-iFluor 594 (1:1,000, Baiao Leibo, Beijing, China) was used to stain F-actin, nuclei were stained with DAPI (Solarbio, Beijing, China), and confocal images obtained using a Zeiss LSM 800 confocal microscope. Image J software was used to measure fluorescence intensity.

### In vivo bio-distribution imaging

The in vivo bio-distribution of NanoFVax was characterized using C-terminal mCherry-tagged vector DNA vaccine. The backs of BALB/c mice were shaved and injected bilaterally with 15 μg of DNA for a total of 30 μg of DNA injected intramuscularly. Images were collected at 4, 8, 12, 24, and 48 h after injection, and fluorescence signals were detected in two mice per group.

Mice were euthanized after the last imaging session and subjected to conventional ex vivo bio-distribution to determine the bio-distribution of the nanoparticles at specific time points. Fluorescence signals were measured using an AMI optical imaging system (Spectral Instruments Imaging Inc., Tucson, AZ, USA; λ ex = 488 nm; λ em = 690 nm; exposure time, 30 s).

### Animal immunization

8-week-old-BALB/c female mice with a mean weight of 20 g were obtained from Liaoning Changsheng Biotechnology (Liaoning, China, License No. SCXK 2020–0001) and maintained according to the regulation approved by the Institutional Animal Ethics Committee of Henan Agricultural University. All the animal studies, we adhered to the 3 R principles (reduction, replacement, and refinement). After a 3-day acclimation period, BALB/c mice were randomly divided into 3 groups (n = 5), and subcutaneously immunized intramuscularly, in the contralateral thigh muscle, with 10 μg per mouse of NanoFVax or monomer on days 0, 14 and 28. PBS was used as a control. Based on our previous study, we used a combination of adjuvants, equal amounts of MF59 and CpG-1826 adjuvants in NanoFVax or monomer group [21]. Serum samples were collected periodically from the tail vein of mice and stored at −80 °C until analysis. On the 42nd day, mice were euthanized using carbon dioxide (CO_2_) in an appropriate euthanasia chamber. Spleens were harvested under sterile conditions and processed into single cells for experimental use.

### Antibody titer and isotype assay

Serum was assayed for antibody titers and subtypes by ELISA. Briefly, the antigen (monomer) was coated onto 96-well plates (300 ng well^−1^) in carbonate buffer (pH = 9.6) at 4 ℃ overnight. After drying, the plates were sealed with 5% BSA in PBS at 37 °C for 1 h. After washing with PBST, 100 μL of diluted serum was added and incubated at 37 °C for 1 h. Thereafter, wells were incubated with HRP-conjugated goat anti-mouse IgG (H + L), IgG1, IgG2a, IgG2b, IgG2c, or IgG3 antibodies (Proteintech, Wuhan, China) at 37 °C for 1 h. The samples were washed and incubated with the chromogenic substrate, TMB (3,3′, 5,5′-tetramethylbenzidine) (Solarbio, Beijing, China), for 15 min at room temperature. The reaction was stopped by adding 2 M H_2_SO_4_ (50 µL well^−1^), and the absorbance was measured at 450 nm using an ELISA flat-panel reader (TECAN, Mannedorf, Switzerland).

### Lymphocyte proliferation and cytokine assay

Splenocyte suspensions were prepared prior to the determination of mouse lymphocyte proliferation. Immunized mice were sacrificed, spleens were dissected from the mice and disrupted in PBS and then placed in RPMI-1640 medium. Splenocytes were crushed and passed through a sterile 70 μm cell sieve into the medium. The cells were centrifuged at 1200 g for 5 min at 4 °C. The erythrocytes were lysed with 5 mL erythrocyte lysis buffer (Solarbio, Beijing, China) for 5 min, and the erythrocytes were removed by washing once with RPMI-1640 medium. Finally, the cells were resuspended in RPMI-1640 medium containing 10% fetal bovine serum and adjusted to a concentration of 1 × 10^6^ mL^−1^. Treated lymphocytes were inoculated into 96-well plates with monomer as stimulant (5 μg mL^−1^), with ConA (Sigma-Aldrich, 10 µg mL^−1^) as positive control and RPMI-1640 medium as negative control. Splenocytes used as stimuli were pre-cultured in an incubator at 37 °C, 5% CO_2_ for 48 h, 3-(4,5-dimethylthiazol-2-yl)-2,5-diphenyltetrazolium bromide (MTT) solution was added to the cells followed by 4 h of incubation, the MTT solution was then carefully removed, and MTT formazan was dissolved by adding DMSO. The mean optical density was measured at 490 nm and the stimulation index was calculated based on the ratio of the growth of the experimental group to the growth of the negative group.

ELISA kits for IL-2, IFN-γ, TNF-α, IL-12 (Invitrogen, Waltham, MA, USA) and IL-4, IL-10 (Ruixin Biotech, Nanjing, China) were used to detect the indicated cytokines in sera according to the manufacturer's instructions. Cytokine concentrations were calculated using standard curves. Cytokine profiles were analyzed using a Spark 20 M plate reader (TECAN, Mannedorf, Switzerland).

### ELISPOT assays

Antigen-specific splenocytes from immunized BALB/c mice were detected using mouse IFN-γ and IL-4 ELIspot kits (DAKEWE, Beijing, China). Briefly, 14 days after the last immunization, three mice were randomly selected from each group and spleens were harvested for ELISpot detection. Mouse splenocytes were isolated in RPMI-1640 medium (Solarbio, Beijing, China) containing 10% FBS (Gibco, Grand Island, NY, USA) and penicillin/streptomycin to prepare a single-cell suspension. Cells were diluted to 2 × 10^6^ with serum-free medium and a volume of 100 μL volume was added to 96-well ELISpot plates. Purified protein was cultured at a final concentration of 10 μg well^−1^. Spots were developed according to the manufacturer's instructions. The number of IFN-γ or IL-4 positive T cells was calculated using CTL-ImmunoSpot^®^ S6 cell reader/ImmunoSpot 7.0.15.1 software (Cellular Technology Limited, Shaker Heights, OH, USA).

### Flow cytometry

The spleens of immunized mice were collected in PBS, ground, and filtered through a 70 μm sterile cell filter to remove clumps. Samples were centrifuged at 1,500 rpm for 5 min, single cell suspensions were prepared, and red blood cells were lysed with red blood cell lysis solution (Solarbio, Beijing, China). Single spleen cells were collected through a 45 μm filter and 2 × 10^5^ cells were placed into Eppendorf tubes. For B and T cell surface marker staining, cells were stained with fluorochrome-conjugated monoclonal antibodies at 4 ℃ for 30 min in the dark. The cells were washed and analyzed by flow cytometry. The following antibodies were used; anti-CD3 APC, anti-CD4 FITC, anti-CD8 FITC, anti-CD19 APC, anti-GL7 PE, anti-IgD PE, anti-CD20 FITC, anti-CD95 FITC, anti-PD-1 APC, anti-CD44 PE, anti-CD25 FITC, and anti-CD69 APC, anti-CD278 FITC, anti-CXCR5 FITC, anti-CD138 FITC, anti-CD27 FITC (BioLegend, San Diego, CA). Flow cytometry and FlowJo software version 10.6.1 (BD Biosciences, San Jose, CA, USA) were used for cell analysis*.*

### Statistical analysis

Data are expressed as means ± standard deviation (SD). Student’s *t*-test was used to determine significance with Graphpad prism 8.0 (GraphPad, CA, USA). Statistical significance was considered and expressed as ^*^p < 0.05, ^**^p < 0.01, and ^***^p < 0.001.

## Results

### Identification of the immune-dominant peptides

Based on ABCpred and PROTEAN (DNAStar, Madison, USA) prediction tools, a full list of B cell epitope prediction results per amino acid position per protein is provided in Table 1 and Additional file 1: Figure S1A–D. To take a further validation, we used dot blot analysis to assess the affinity of ASFV-positive serum to the synthesized peptides. The dot blot assay inferred that the peptide sequences including pB602L ^39^FKNDSRVAF^47^, pB602L ^87^TTKTLLSEL^95^, pB602L ^101^TLKQETNDVPSES^113^, p30 ^16^KTDLRSSSQV^25^, p30 ^75^TEHQAQEEWNMI^86^, p72 ^81^TGTPTLGNKLTFGIP^95^, p72 ^279^HFPENSHNIQTA^290^, CD2v ^34^NDNNDINGVSWNF^46^, CD2v ^84^IFPHNDVFDTTYQ^96^ can bind to ASFV-positive serum (Additional file 2: Figure S2A). In addition, the immunodominant B cell epitopes (pB602L ^474^SKENLTPDE^482^, p72 ^221^MTGYKH^226^, CD2v ^160^WNNSNINNFT^169^) from our previous studies were added to the next step of immunogen design [22].

To select T cell epitopes, we first screened two structural proteins, CD2v and p72, for CD8 T cell epitopes using NetMHC 4.0 software, which predicts peptide binding rankings for peptide synthesis with class I SLAs (Table 2). In order to determine which epitope (s) could induce T cell response, the ELISpot was conducted. 14 days following the last boost, mouse splenocytes were re-stimulated ex vivo by the peptides. In accordance with the predictions, only two epitopes, p72 SLA-1*0401 ^522^ISDISPVTY^530^ and CD2v SLA-1*0401 ^150^YTNESILEY^158^, were recognized by CD8 + T cells (Additional file 2: Figure S2B).

Highly conserved dominant epitopes provide the advantages of high immunogenicity, broad-spectrum protection, and resistance to escape mutations for ASFV vaccine design. Next, we analyzed the sequence conservation of predicted epitopes using ESPript3.0 software. We mainly compared p72, CD2v, pB602L and p30 protein sequences of different ASFV genotypes, including Chinese ASFV strain (GenBank No. MK333180.1) as the reference strain, and analyzed the eleven identified peptide sequences. The results showed that the immunodominant B-cell epitopes p72 ^81^TGTPTLGNKLTFGIP^95^, p72 ^279^HFPENSHNIQTA^290^, CD2v ^34^NDNNDINGVSWNF^46^, CD2v ^84^IFPHNDVFDTTYQ^96^, pB602L ^39^FKNDSRVAF^47^, pB602L ^87^TTKTLLSEL^95^, pB602L ^101^TLKQETNDVPSES^113^, p30 ^16^KTDLRSSSQV^25^ and p30 ^75^TEHQAQEEWNMI^86^ were highly conserved in different ASFV strains, and the T-cell epitopes p72 SLA-1*0401 ^522^ISDISPVTY^530^ and CD2v SLA-1*0401 ^150^YTNESILEY^158^ were also highly conserved among all strains (Additional file 3: Figure S3).

**Fig. 1:**
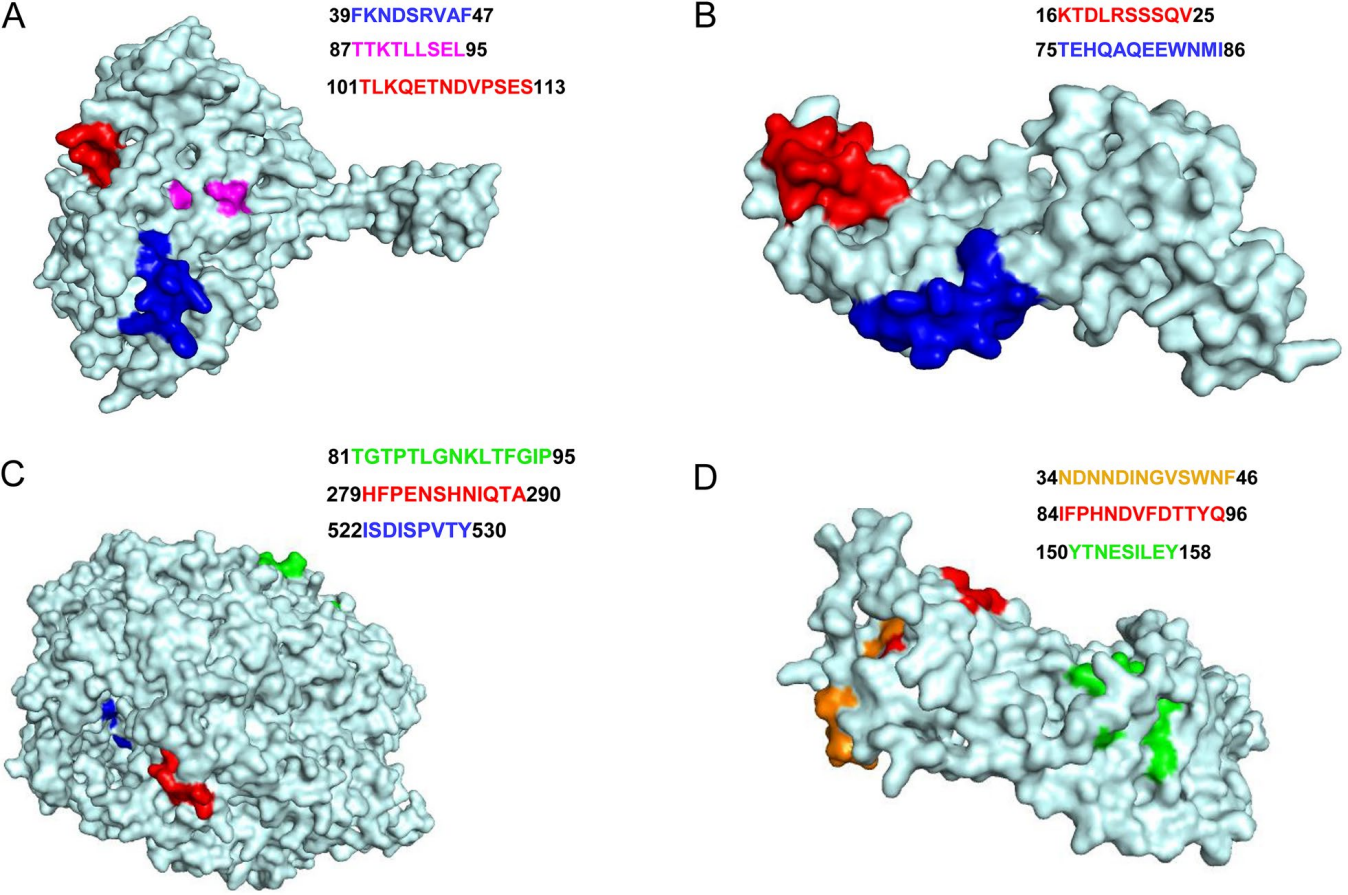
Structural feature of epitope peptides. **A**–**D** are protein structural predictions of ASFV pB602L, p30, p72, and CD2v, respectively. All images displayed in surface mode and colored in cyan. The epitope regions are shown in blue, red, green, and orange

Nine epitopes were located on the surface of pB602L, p30, p72 and CD2v proteins, while a few amino acids of pB602L ^87^TTKTLLSEL^95^ and CD2v ^34^NDNNDINGVSWNF^46^ are likely to be located in the interior of the protein (Fig. 1). Thus, the epitopes selected in this study are highly conserved and provide a viable strategy and option for the next step in the design of ASF vaccine candidates. Therefore, the favorable epitopes selected in this study provide a valuable basis for future research and development of potentially effective vaccines against ASFV.

### Nanoparticle vaccine construction and purification

**Fig. 2:**
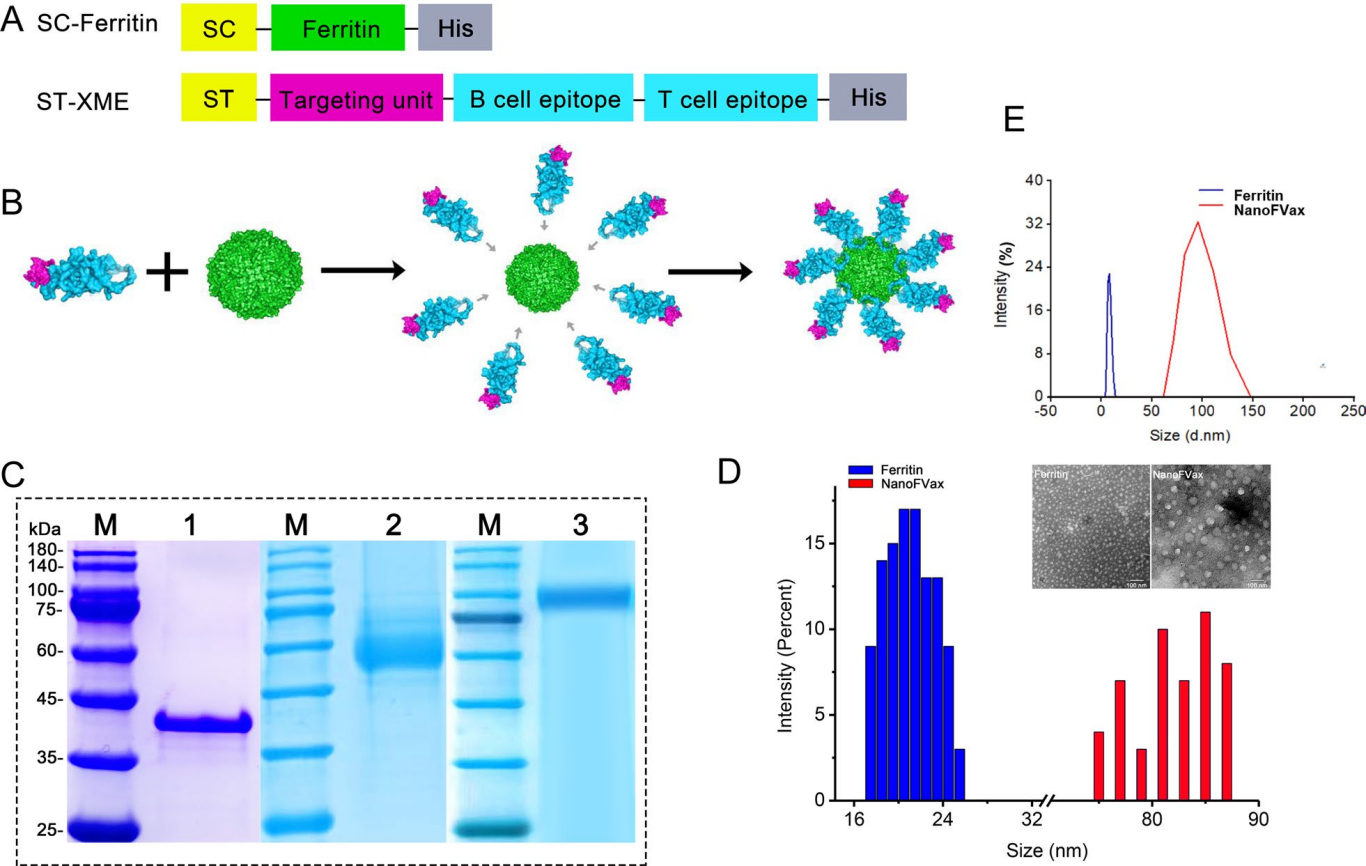
Schematic and characterization of the multi-epitope nanoparticles prepared with the SpyTag-SpyCatcher ligase system. **A** The ferritin sequence was ligated to the C-terminus of SpyCatcher and contained a 8 × His-tag at the C-terminus. The targeting unit (XCL1) was ligated to the C-terminus of SpyTag and the B-cell and T-cell epitopes ligated to the C-terminal of the targeting unit and to each other in tandem using a flexible linker (GGGS). The chimeric sequences were inserted into into pET28a ( +) between the BamHI and XhoI sites. SC: SpyCatcher. ST: SpyTag. **B** The 3D schematic diagram of nanoparticles. The magenta color represents the targeting molecule, the blue color represents the tandem multi-epitope, and the green color represents ferritin; **C** SC-Ferritin (lane 1), ST-XME (lane 2), and NanoFVax (lane 3) validated by SDS-PAGE analysis, lane M, protein marker (New Cell & Molecular Biotech, Suzhou, China). **D** Observation of ferritin nanoparticles by TEM, ferritin (left) and NanoFVax (right). **E** Size distribution of nanoparticles before and after conjugation by dynamic light scattering (DLS)

In order to improve the expression of two different protein subunits, we employed the SpyTag/SpyCatcher system. This system originated in *Streptococcus pyogenes* for covalent coupling of ferritin-based nanoparticles. Here, we used ASFV multi-epitope ferritin nanoparticles as an antigen delivery platform (Fig. 2A). The 3D structure and assembly diagram of NanoFvax are predicted using AlphaFold, as shown in Fig. 2B. Ferritin nanoparticles (PDB access No. 3EGM). To increase the protein solubility, co-expression was accomplished with the molecular chaperone pTF16 plasmid. SDS-PAGE analysis demonstrated that the SC-Ferritin and ST-XME fusion proteins were approximately 35 kDa (Fig. 2C, lane 1) and 65 kDa (Fig. 2C, lane 2), respectively. Purified SC-Ferritin and ST-XME were mixed at a 1:24 molar ratio and incubated overnight at 4 °C in PBS buffer. SC-Ferritin and ST-XME formed an intermolecular iso-peptide bond that binds ferritin to the antigenic subunit. As SDS-PAGE results showed that the molecular weight of the coupled protein was higher than that of the theoretical monomer protein, the coupled proteins obtained were of the expected molecular weight (NanoFVax: 100 kD) (Fig. 2C, lane 3), indicating that the two proteins were successfully ligated by spvtag/spycatcher. The immunobloting was performed under non-reducing conditions (without DTT). Further purification by nickel-chelate affinity chromatography and performed LAL detection, the lower detection limit (LOD) for endotoxin was 0.005 EU/mL. TEM results showed the spherical morphology of ferritin and NanoFVax (Fig. 2D). DLS analysis showed that in PBS solution, the Z-average diameter of NanoFVax nanoparticles was 86.10 nm, while ferritin had a Z-average diameter of approximately 20 nm. Both TEM and DLS data showed that the purified samples were had a uniform size and were well dispersed.

### Nanoparticles elicit BMDC maturation in vitro

**Fig. 3:**
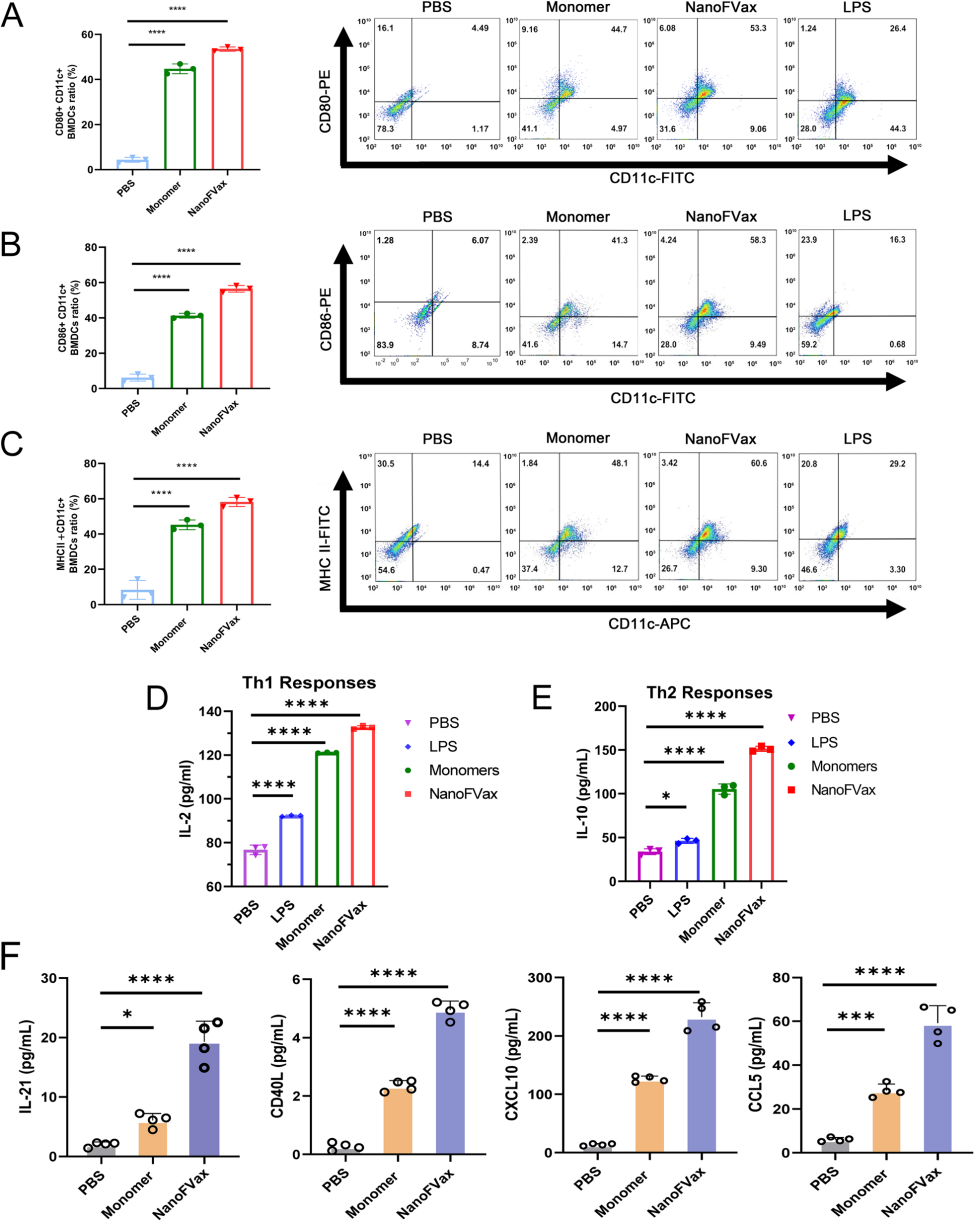
NanoFVax elicited BMDCs maturation in vitro. CD80 + (**A**), CD86 + (**B**), and MHC-II + (**C**) expression on mDCs determined by flow cytometry after co-incubation with different stimulants for 48 h. The bar graphs show the statistics for CD80 + (**A**), CD86 + (**B**), and MHC-II (**C**). Comparison of cytokines and chemokines in BMDC supernatants of each group. Culture supernatants were analyzed for levels of the Th1 cytokine, IL-2 (**D**), the Th2 cytokine, IL-10 (**E**) (n = 3), and **F** IL-21, CD40L, and the chemokines CXCL10, CCL5 as measured by ELISA (n = 4). ^*^p < 0.05, ^**^p < 0.01, ^***^p < 0.001, ^****^p < 0.0001 by Student’s *t*-test

BMDC maturation and antigen presentation capacity were assessed by flow cytometry and ELISA assay. The cell surface expression of the co-stimulation molecules CD80 + , CD86, and MHC II were detected using flow cytometry, under stimulation. Commercial LPS was used as a positive control. After treatment with NanoFVax for 48 h, the percentage of BMDCs that were CD80 + (53.5%) and CD86 + (58.3%) was significantly higher than that of CD80 + (44.7%) (Fig. 3A) and CD86 + (41.3%) in the monomer groups (Fig. 3B). Nanoparticles also increased the expression of MHC II in BMDCs (60.6%) (Fig. 3C), indicating an enhanced presenting capacity of these BMDCs. Consistent with the flow cytometry data, cytokine levels in BMDC supernatants harvested after 48 h of stimulation showed similar results. IL-2 and IL-10 play important roles in regulating T-cell proliferation and function. As shown in Fig. 3D, E, respectively, the levels of IL-2 and IL-10 were significantly increased in NanoFVax-treated BMDCs.

We also evaluated the secretion of IL-21, CD40L, CXCL10 and CCL5 in the supernatant, with the NanoFVax-treated BMDCs inducing the highest secretion levels of these molecules (Fig. 3F). Compared with the monomer and PBS groups, the secretion of IL-21, CD40L and CXCL10 was twofold higher in the NanoFVax-treated group, while the secretion of CCL5 was 2–threefold higher. These results demonstrated that NanoFVax can not only stimulate DC maturation, but also enhance the antigen presentation ability of DC, which are important prerequisites for the induction of T-cell immune and activating inflammatory responses.

### Cellular uptake and bio-distribution studies

**Fig. 4:**
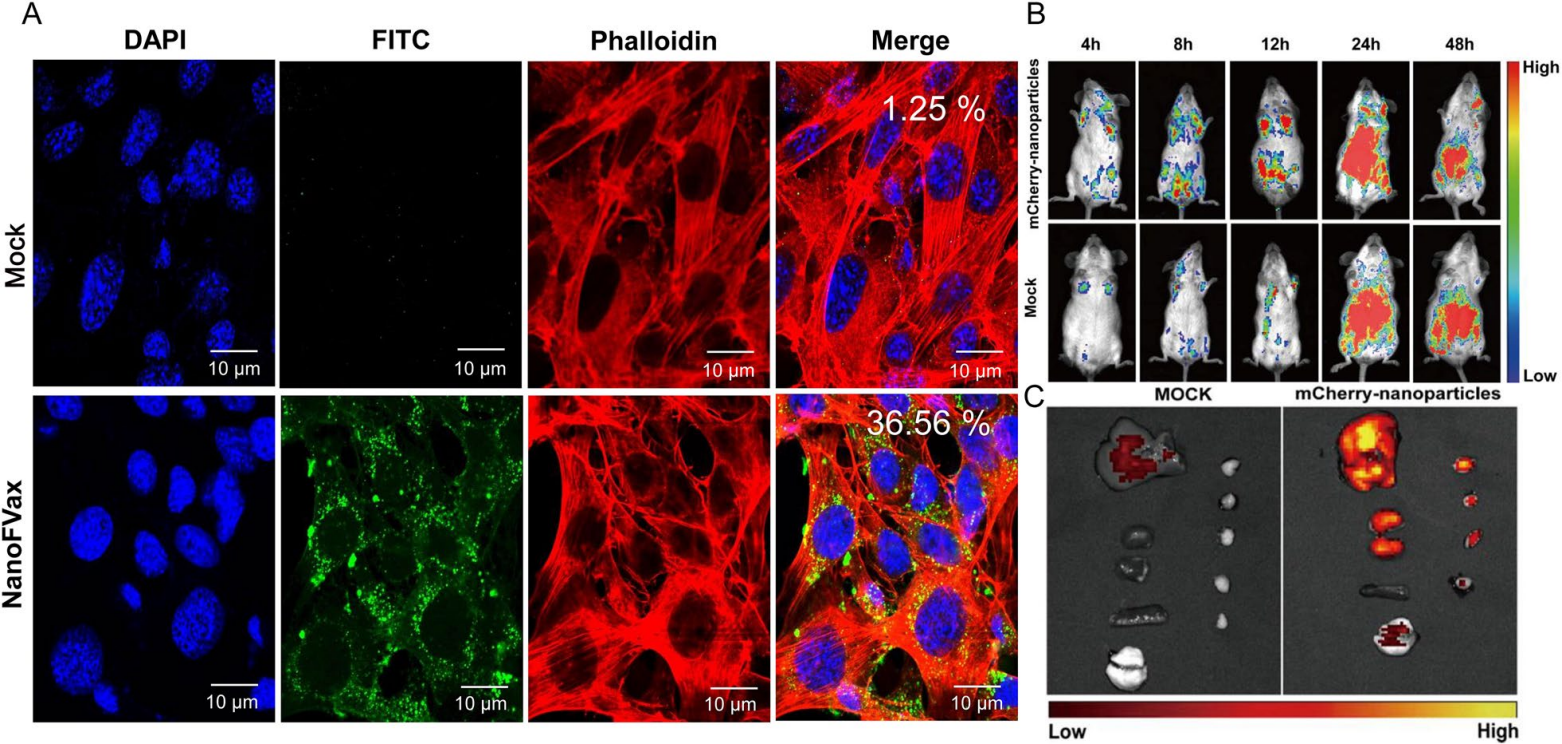
Confocal images of cellular uptake and in vivo bio-distribution of NanoFVax. **A** Visualization of cellular uptake of fluorescein isothiocyanate (FITC)-labeled NanoFVax-treated DC cells after 24 h. Numbers indicate relative intensity as quantified by Image J analysis. DAPI staining (blue) was used to visualize nuclei. Actin cytoskeleton was visualized with phalloidin-iFluor 594. Free FITC was used as a control. Numbers indicate relative intensity as quantified by image J analysis. **B** Fluorescence imaging of mice of the different injection groups. Representative images of one mouse from the two mice per group are shown. **C** Ex vivo fluorescence imaging of major organs 48 h after subcutaneous injection. (The first column is the lungs, kidneys, spleen, and heart from top to bottom; the second column is the lymph nodes)

As the most potent APC, DCs play a critical role in the induction of protective immunity. Antigen internalization is an important prerequisite for subsequent DC activation and antigen cross-presentation. To determine whether the NanoFVax can be recognized and processed by the host immune system, we performed cellular uptake experiments. After 24 h co-incubation of FITC-labeled NanoFVax with mouse dendritic cells (DC2.4), laser scanning confocal microscopy showed a significant enhancement of the fluorescence signal, with the green fluorescence mainly distributed in the cytoplasm. These results indicate that NanoFVax can be rapidly taken up and internalized by DCs. Taken together, the nanoparticles could be effectively phagocytosed by DCs, resulting in more efficient antigen presentation in lymph nodes (Fig. 4A).

Previous reports have shown that nanoparticle vaccines were efficiently shed with accumulation in lymph nodes, thereby enhancing subsequent interactions with APCs and B cells. To validate the distribution of nanoparticles in vivo, we constructed recombinant mCherry-tagged nanoparticle vectors for antigen tracking and bio-distribution studies. After injection of mCherry-tagged DNA, the fluorescence signal began to decay at 48 h and was significantly weaker than at 24 h (Fig. 4B). The intake of the NanoFVax was significantly greater than that of the control group, which was consistent with the results of in vivo fluorescence imaging. Tissues (heart, liver, spleen, lung and kidney) from immunized mice were then analyzed by in vitro imaging. Strong fluorescent signals were found in the lungs, lymph node, and kidneys at 48 h. Overall, the uptake of NanoFVax was significantly high than that of the control group, consistent with the results of in vivo fluorescence imaging in the lungs, lymph node, and kidneys at 48 h (Fig. 4C).

### Nanoparticles elicit a robust and durable humoral response

**Fig. 5:**
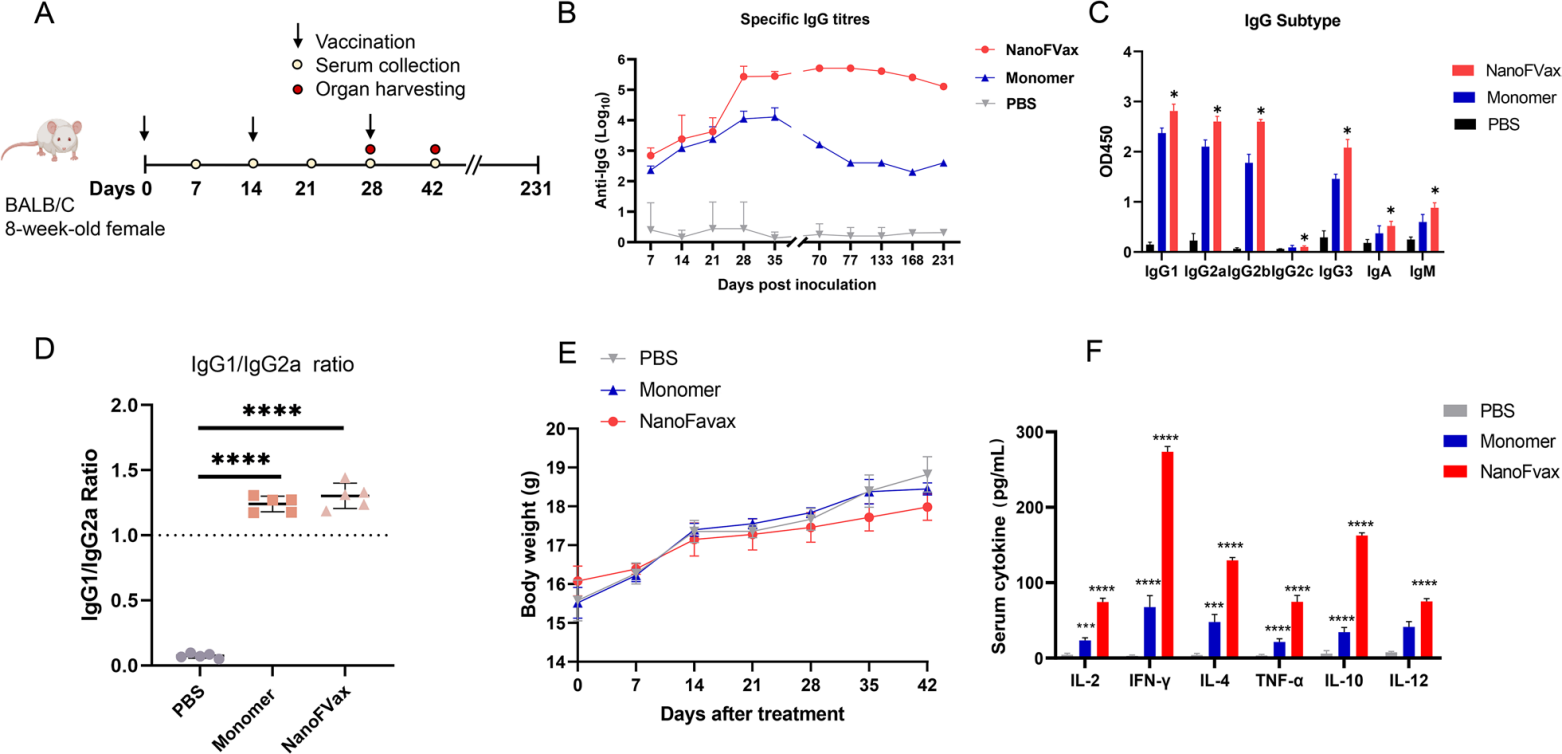
Diagram illustrating the experimental schedule. **A** BALB/C mice were immunized according to the immunization procedure. Five mice per group were vaccinated with different vaccines on days 0, 14, and 28. Serum was collected periodically. Three mice were euthanized on days 42. **B** Serum IgG antibody titers were determined by the serial dilution method, with specific IgG titers calculated for each sample and plotted as time course curves expressed as the reciprocal of the endpoint serum dilution. Serum IgG titers were transformed to log_10_. Thus, all concentrations were log_10_ transformed prior to statistical analysis. (Mean log_10_ IgG titers = 5.1). **C** Serum was collected from mice and Ig (IgG1, IgG2a, IgG2b, and IgG2c, IgA, IgM,) levels were measured on days 42 as well as the IgG1/IgG2a ratio (**D**). **E** Body weight changes of treated mice following intramuscular injection (n = 5). **F** Serum cytokines (IL-2, IFN-γ, IL-4, TNF-α, IL-10, and IL-12) were assayed for each group 42 days after immunization. Data are the mean of three independent experiments. Mean values and error bars are defined as means and S.D., respectively. Student’s *t*-test. ^*^p < 0.05; ^**^p < 0.01; ^***^p < 0.0001; ^****^p < 0.00001

To assess the immune response to nanoparticles and monomer, mice were immunized with 10 μg/mouse NanoFVax or monomer (with equal amounts of MF59 and CpG-1826 adjuvants, n = 5 in three replicate immunizations) (Fig. 5A). To evaluate the specificity of the antibodies produced by NanoFVax or monomer, we performed ELISA with recombinant monomer protein as coating antigen. PBS-immunized mice sera were used as control. The greatest specific antibody titres were induced by the nanoparticle vaccine at 14 days (at the time of the third vaccination), and were significantly higher than those that in the other groups (Fig. 5B). The monomer vaccine also induced specific antibodies at 28 days, but the antibody level decreased significantly after 35 days. Moreover, we found that the antibody titer after NanoFVax injection remained at a high level up to 133 days, and then decreased slightly until to 231 days. These results suggest that the NanoFVax elicited a stronger response than the monomer vaccine as judged by both antibody titer and antibody duration.

To assess the polarization of the immune response, we quantified IgG2a, IgG2b, IgG2c, IgG3, IgA and IgM titers in the sera of mice on day 14 after triple immunization. IgG1-biased antibody responses were induced, and significant levels of antigen-specific IgG2a, IgG2b, IgG3 were detected in the sera of mice (Fig. 5C). It is a known fact that the ratio of IgG1/IgG2a was used as an index of a Th1/Th2 mediated response. As shown in (Fig. 5D), NanoFVax-immunized mice had more abundant specific IgG1 and IgG2a, with an IgG1/IgG2a ratio greater than 1 and higher than that of the other groups, indicating a greater Th1 and Th2 immune response. The body weight of mice immunized with NanoFVax showed a negligible change compared to the control group (Fig. 5E). The results showed that a 10 mg dose of NanoFVax elicited a stronger and longer lasting immune response than the monomer and prompted us to evaluate its prophylactic potential in ASFV.

### Serum cytokines

Forty-two days after immunization, the secretion of cytokines in serum were measured by ELISA. The secretion of IL-2, IFN-γ, IL-4, TNF-α, IL-10 and IL-12 were found to be significantly increased by NanoFVax immunization. These results demonstrated the vaccine can elicit a hallmark cellular immune response, and further suggested that the immune cells activated in vivo are biased toward a type 1 cellular immune response accompanied by a type 2 cellular immune response (Fig. 5F).

### Nanoparticles stimulate the activation of T follicular helper cell (TFH) and B cell

**Fig. 6:**
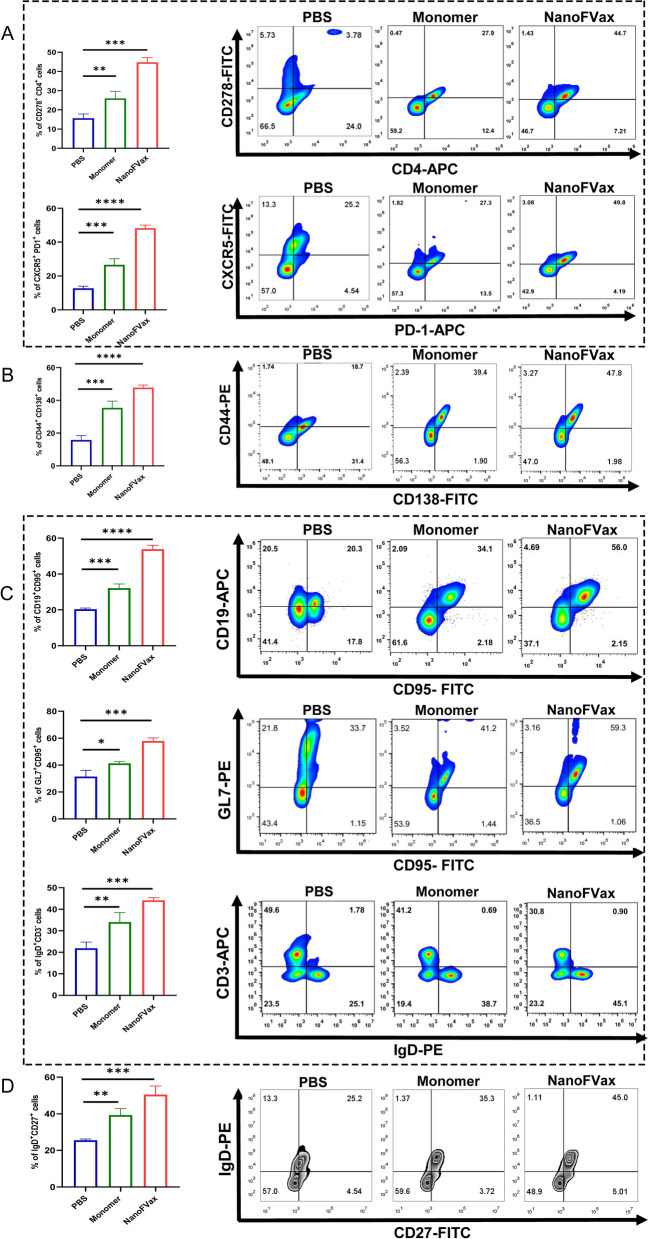
NanoFVax induces robust T_FH_ cell response and T-/B-cell coordination. BALB/c mice were immunized with different vaccines. After the second immunization on the 14th day, the mice were euthanized then assessed by flow cytometry. **A** The percentage of T_FH_ cells (CD278 + CD4 + CXCR5 + PD-1 +), **B** Plasma cells (CD44 + CD138 +), **C** GC B cells (CD19 + CD95 + GL7 + IgD +) and **D** M B cells (IgD + CD27 +) were determined (n = 3). Representative histogram. Experiments were performed independently in triplicate. Data are expressed as mean ± SD, *p* values were calculated by Student's *t*-test. Statistically significant differences were at *p < 0.05, **p < 0.01, ***p < 0.001

In addition to the above serological responses, we also examined the percentages of different lymphocytes. We found that the percentages of T_FH_ (CD278 + CD4 + CXCR5 + PD-1 +) (Fig. 6A), plasma cells (CD44 + CD138 +) (Fig. 6B), germinal center (GC) B cells (CD19 + CD95 + GL7 + IgD +) (Fig. 6C) and memory B (M B) cells (IgD + CD27 +) (Fig. 6D) in the spleens of nanoparticle-vaccinated mice were significantly higher than that in monomer–immunized mice. These results suggest that the nanoparticle vaccine induced higher levels of T_FH_ and promoted B cell activation, and the synergistically promoted B cell maturation. Taken together, our results suggest that antigens associated with NanoFVax are more efficiently antigen-presenting and T-B cell synergistic compared to monomer.

### Nanoparticles enhance T cell immune responses

**Fig. 7:**
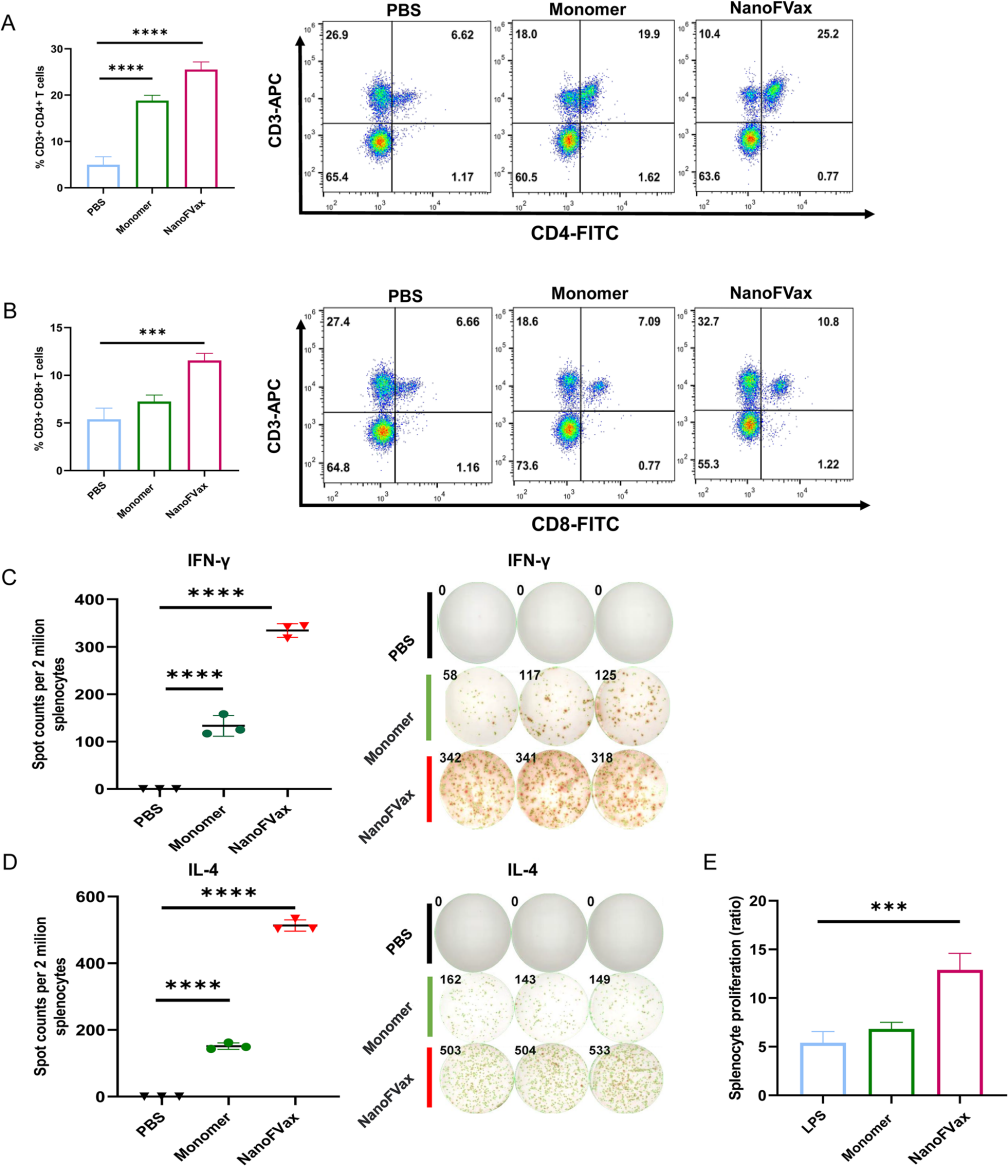
NanoFVax enhanced cellular immune responses in vivo. Percentage of splenic CD4 + (**A**) or CD8 + (**B**) T cells determined by flow cytometry. The response of different groups after in vitro stimulation, IFN-γ (**C**) and IL-4 (**D**) spot-forming cells and statistical analysis. Images were acquired and quantified using an ELISpot reader. **E** Cell proliferation was detected by MTT assay. NanoFVax induced significant cell proliferation compared to control. (n = 3), the data in 5A-5C are shown as the mean and standard deviation (mean ± SD). ^*^p < 0.05; ^**^p < 0.01; ^***^p < 0.001, ^****^p < 0.0001

T cell immunity has been shown to play a critical role in combating ASFV infection. On the 14th day after immunization, the proliferation of CD3 + T cells in the spleens of immunized mice and their differentiation into CD4 + and CD8 + T cells in vivo were evaluated by flow cytometry. Compared to PBS and monomer groups, the amount of CD3 + CD4 + T cells activated by NanoFVax was significantly increased (*p* < 0.001) (Fig. 7A). In addition, the CD8 + T cells induced by NanoFVax were greater than those induced by monomeric antigen and PBS (*p* < 0.05) (Fig. 7B). Furthermore, according to the detection results, it can be concluded that the cellular immunity induced by NanoFVax is more inclined to helper T cell immunity, and CD4 + T cells are more than CD8 + T cells. Collectively, NanoFVax was found to elicit both CD4 + and CD8 + T-cell responses.

ELISpot was used to further analyze the effect of NanoFVax on T cells by analyzing the secretion of IFN-γ and IL-4 in the supernatants of the stimulated PBMCs. Notably, PBMCs pretreated with NanoFVax induced a greater proportion of IFN-γ (Fig. 7C) and IL-4 (Fig. 7D) secreting cells than the monomer group. In addition, we used two stimulants, NanoFvax and monomer, to assess the proliferative activity of mouse lymphocytes (Fig. 7E). The results showed that when stimulated with the positive control, LPS, the proliferative capacity of lymphocytes in all experimental groups was higher than in the control group. In addition, the proliferative capacity of lymphocytes stimulated by NanoFvax group was stronger than that stimulated by monomer (*p* < 0.05). Taken together with the above ELISpot and lymphocyte proliferation analysis, these results suggest that NanoFvax induces a potent ASFV-specific cellular immune response.

## Discussion

In the absence of a vaccine, ASF poses a serious threat to the global swine industry. A safe and effective vaccine is urgently needed to control the spread of ASF. Currently, vaccine development for ASF is severely hampered by limitations in the understanding of the structure and function of the proteins encoded by ASFV, as well as the complexity of the viruses. Previous studies have focused primarily on subunit vaccines [23].

The p72, pB602L, p30 and CD2v proteins are promising targets for vaccine development, which are highly immunogenic based on previous studies [1, 24]. Several studies have shown that p72 and CD2v contain many cross-reactive CD4 + and CD8 + T cell epitopes that may induce a neutralizing immune response [11, 25].

*Helicobacter pylori* ferritin self-assembles into 24-subunit particles that display eight trimeric antigens on a surface triploid axis [26]. *H. pylori* ferritin has not been reported to typically induce antibodies, so it is therefore unlikely to do so [27]. Therefore, we chose ferritin as the core of the nanoparticle vaccine. SpyCatcher/SpyTag is a very versatile tool used in many applications to connect translated proteins by forming irreversible covalent bonds [28]. This tool has been widely used for basic research and biotechnology applications [29, 30].

In this study, ferritin was used as a nanocarrier to enhance vaccine efficacy. We prepared a nanoparticle vaccine candidate targeting XCL1 on classic dendritic cells (cDC1s), and containing immunodominant ASF B- and T-cell epitopes (NanoFVax). After three immunizations with NanoFVax, all experimental mouse groups induced a significant increase in serum IgG antibodies and exhibited a durable humoral immune response.

Previous reports have shown that nanoparticle vaccines were effectively phagocytosed by APCs that accumulated in lymph nodes, enhancing immune processing. The T_FH_ cell help is essential for B-cell activation and GC formation [31]. Nanoparticle vaccines are readily taken up by DCs and macrophages, thereby promoting coordination between T_FH_ (T follicular helper cell) and B cells, with T_FH_ the most important for production of antibodies by B cells [32–34]. Importantly, we found that the percentage of T_FH_, plasma cells, GC B cells and MBC in the spleens of NanoFVax-vaccinated mice were significantly greater than those of monomer-vaccinated mice. In conclusion, these data demonstrate the potent stimulatory effect of NanoFVax on T_FH_ differentiation and B cell responses. cDC1 recruit and activate T cells and as such DC-based vaccines are promising because of their ability to elicit broad and long-lasting immune effects. As a result, nanoparticle vaccines were found to be superior to monomeric protein with regard to both humoral and cellular immune responses.

CXCL10 is a T cell and monocyte chemoattractant induced by both type I and type II IFNs that direct T cell chemotaxis through activation of CXCR3, a G protein-coupled 7 transmembrane receptor [35]. Furthermore, CXCL10 regulates the development and function of T cells, recruiting Th cells expressing the chemokine receptor CXCR3 (primarily Th1 cells), and stimulating the activation and migration of immune cells (such as NK cells, monocytes, and T cells) to sites of infection [36, 37]. CD40L is a member of the tumor necrosis factor superfamily (TNFSF) widely expressed on activated CD4 + T cells, promoting B cell maturation and antibody isotype switching [38, 39]. In its membrane-bound form, CD40L is predominantly expressed on activated CD4 + T cells, whereas its receptor CD40 is expressed on immature DCs, B cells, and other immune and non-immune cells [40]. By targeting CD40 to immature DCs, CD40L induces maturation and activation of DCs, which in turn induce activation of cytotoxic CD8 + T cells [41, 42]. CD40L binding of CD40 to B cells has been shown to promote B cell proliferation and survival, antibody isotype switching, and antibody affinity maturation [43]. CCL5 is a chemokine ligand for CCR5, CCR3, and CCR1 receptors found on activated T cells, natural killer cells, immature DCs, and other cells [44]. Notably, we observed a significant increase in CXCL10, CD40L, and CCL5 levels in the BMDC (Bone marrow derived dendritic cell) supernatants treated with NanoFVax in vitro. In addition, we examined a key indicator of the paracrine response supporting humoral immunity, IL-21, a cytokine secreted by CD4 + T_FH_ cells that regulates the development of memory B cells. IL-21 elicits long-lasting antibody levels, which are important to vaccine development. The results showed that, compared to the monomeric vaccine, the nanoparticle vaccine could rapidly induce specific IgG antibodies that persisted for at least 231 days. The vaccine also induced large numbers of specific IgG1, IgG2a, and IgG2b memory B cells. Although the ferritin 24 mer core also induced ferritin-specific antibodies, the titer of viral specific antibodies was not affected. In addition, we found that nanoparticle vaccines induced higher secretion of IFN-γ, TNF-α, and IL-10 compared to monomeric protein, suggesting that nanoparticle vaccines activate a robust T cell immune response. Furthermore, the nanoparticle vaccine induced a Th2-biased immune response and a slightly higher Th1-biased immune response. The enhanced Th1 response reinforced killing effects for pathogens, clearing potentially infected cells. Moreover, we observed NanoFVax significantly upregulated the expression of cell-surface molecules of T_FH_ cells (CD278 + CD4 + CXCR5 + PD-1 +), plasma cells (CD44 + CD138 +), GC B cells (CD19 + CD95 + GL7 + IgD +) and memory B cells (IgD + CD27 +). All these results indicated that the nanoparticle vaccine candidate showed great potential to induce humoral and cellular immunity.

Cellular immunity plays a key role in vaccine induced production of memory and effector T cells that provide long-lasting immune protection [45]. Mammalian cellular responses to viral infections involve activation of the innate and adaptive immune systems [46]. T cells play a central role in various viral infections, with CD4 + T cells mediating antibody production by B cells and coordinating the responses of other immune cell types, as well as directly initiating the immune response to infectious agents [47]. CD4 + T cells differentiate into Th2 and Th1 cells that drive the adaptive immune response, while CD8 + T cells directly exert cytotoxic activity, causing T lymphocytes to attempt to increase their cytotoxic activity by increasing CD8 protein [48]. CD8 + T cells target infected cells and clear infection sites, mainly through perforin and granzyme as well as the FasL pathway [49]. Our results showed that the humoral and cellular immune response induced by nanoparticle vaccines was superior to that of monomer vaccines. It is also worth noting that NanoFVax was superior to monomer in the generation of antigen-specific CD4 + and CD8 + T cells that readily produce TNF-α and IFN-γ. Both TNF-α and IFN-γ are known Th1 cytokines that activate the effector function of macrophages, neutrophils and CD8 + T cells. Importantly, NanoFVax enhanced activated CD4 + T lymphocytes that contributed to the generation of specific antigen memory CD8 + T lymphocytes compared to the monomeric vaccine. In addition, the processed antigen was able to induce IFN-γ specific CD8 + T cells. The production of IFN-γ and IL-4 by antigen-stimulated PBMCs was used as an indicator of vaccine immunogenicity [50]. After restimulation of immunized splenocytes, the IFN-γ and IL-4 ELISpot response of the nanoparticle group was 2.5 times greater than that of the monomer vaccine group and much greater than that of the control group. In addition, the immunostimulatory factors IL-2, IFN-γ and TNF-α were significantly increased in the peripheral blood of nanoparticle-immunized mice. These results indicated that the nanoparticles effectively induced T-cell activation.

Epitope vaccines have a better safety profile than conventional live attenuated vaccines. A large number of epitope vaccines have been tested and have shown positive effects [51, 52]. In addition, the ability of epitope vaccines to cope with viral strain mutations is much better than that of conventional vaccines. The selection of antigenic epitopes is a key factor in determining the immunogenicity of epitope vaccines. To improve immune efficacy and reduce the risk of adverse immune responses, an ideal epitope vaccine design should include as many immunodominant epitopes as possible. The nanoparticles designed in this study compensated for the lack of immunogenicity of epitope vaccines. The immunodominant multi-epitope nanoparticle vaccine candidate targeting DC shown here had strong immunogenicity and significantly enhanced T and B cell immune responses. In addition, the preparation of these nanoparticles was very fast and easy because the nanoparticles were easily solubilized and could be expressed in large quantities in *E. coli.* Therefore, these nanoparticle vaccines deserve further evaluation in clinical trials.

As ASFV continues to spread, there is an urgent need for safe and effective vaccines or therapeutics. Isolation and culture of ASFV must be performed in Biosafety Level 3 (BSL-3) facilities, which limits vaccine and drug development. Antibody concentration and persistence may reflect the ability of the host to prevent viral infection. Although we evaluated nanoparticle vaccines for strong immunogenicity and persistent antibody responses in mice, we did not evaluate nanoparticle vaccines for protection against ASFV infection in pigs due to time and resource constraints. This study has important implications for ASFV vaccine development, but further investigation is needed to determine the actual mechanism of protection.

## Conclusion

In this study, we constructed a self-assembled nano-ASF vaccine candidate targeting DC cells. These nanoparticles covalently coupled the self-assembled 24-mer ferritin with the dominant B- and T-cell epitopes of ASFV and fused the chemokine receptor XCL1 (a dendritic cell targeting molecule) through the SpyTag/SpyCatcher protein ligase system. Compared to monomeric protein, nanoparticle vaccines induced stronger T and B cell immune responses in mice. The high level of antibody response against ASFV persisted for more than 231 days. Therefore, NanoFVax nanoparticles are an effective and safe vaccine candidate against ASF.

### Supplementary Information

**Additional file 1: Figure S1.** Bioinformatic analysis of epitopes. A-D are the secondary structures and antigenicity predictions of ASFV pB602L, p30, p72, and CD2v respectively, respectively, obtained from the DNAStar Protean program.

**Additional file 2: Figure S2.** Identification of specific B- and T-cell epitopes. (A). Dot blot analysis. The specificity of the peptide sequences was verified by ASFV positive serum. (B). Specific T cell immune response in immunized mice. BALB/c mice were injected intramuscularly with recombinant protein (p72 or CD2v) (dose = 30 μg per mouse for each injection). ELISpot assay for IFN-γ secretion by splenocytes after stimulation of the peptide pool 14 days following immunization. Plot numbers in the symbols are consistent with those listed in Table 1 and Fig. 1.

**Additional file 3: Figure S3.** Epitopes are conserved in several pandemic strains of ASFV. A-D are sequence conservation analyses of ASFV pB602L, p30, p72, and CD2v, respectively. Identical colors indicate an exact match of amino acid residues. Homologous regions of identified protein epitopes are indicated by black dashed boxes.

## Data Availability

All data needed to evaluate the conclusions in this paper are present in the paper or the Supplementary Materials. Additional data related to this paper may be requested from the authors.
