# Supplementary figures and images for: A candidate nanoparticle vaccine comprised of multiple epitopes of the African swine fever virus elicits a robust immune response
Source: J Nanobiotechnology. 2023 Nov 14;21:424. doi: 10.1186/s12951-023-02210-9 (PMC10647103; doi:10.1186/s12951-023-02210-9)

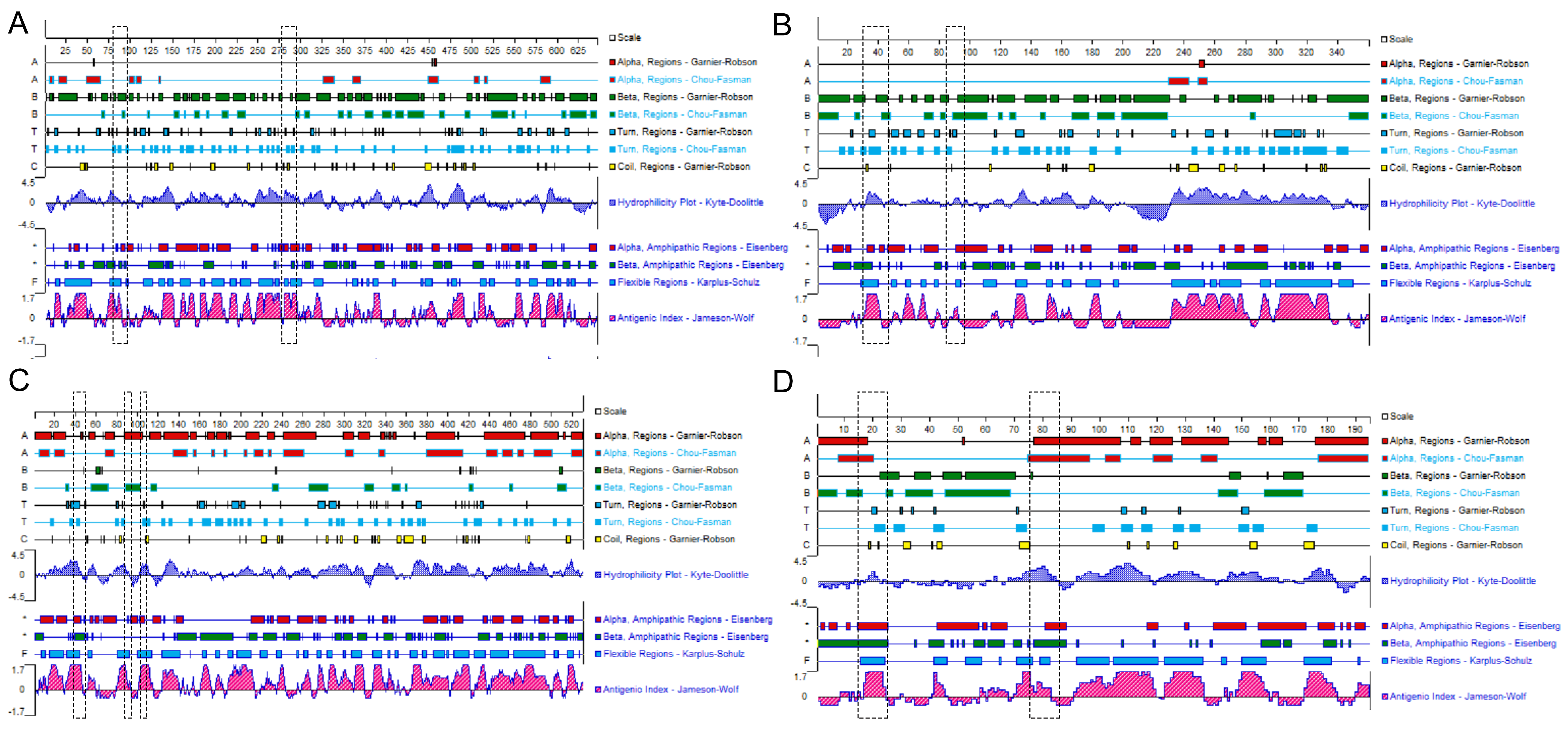

Supplement: Supplementary file 1 — Additional file 1: Figure S1. Bioinformatic analysis of epitopes. A-D are the secondary structures and antigenicity predictions of ASFV pB602L, p30, p72, and CD2v respectively, respectively, obtained from the DNAStar Protean program. [file 12951_2023_2210_MOESM1_ESM.tiff]

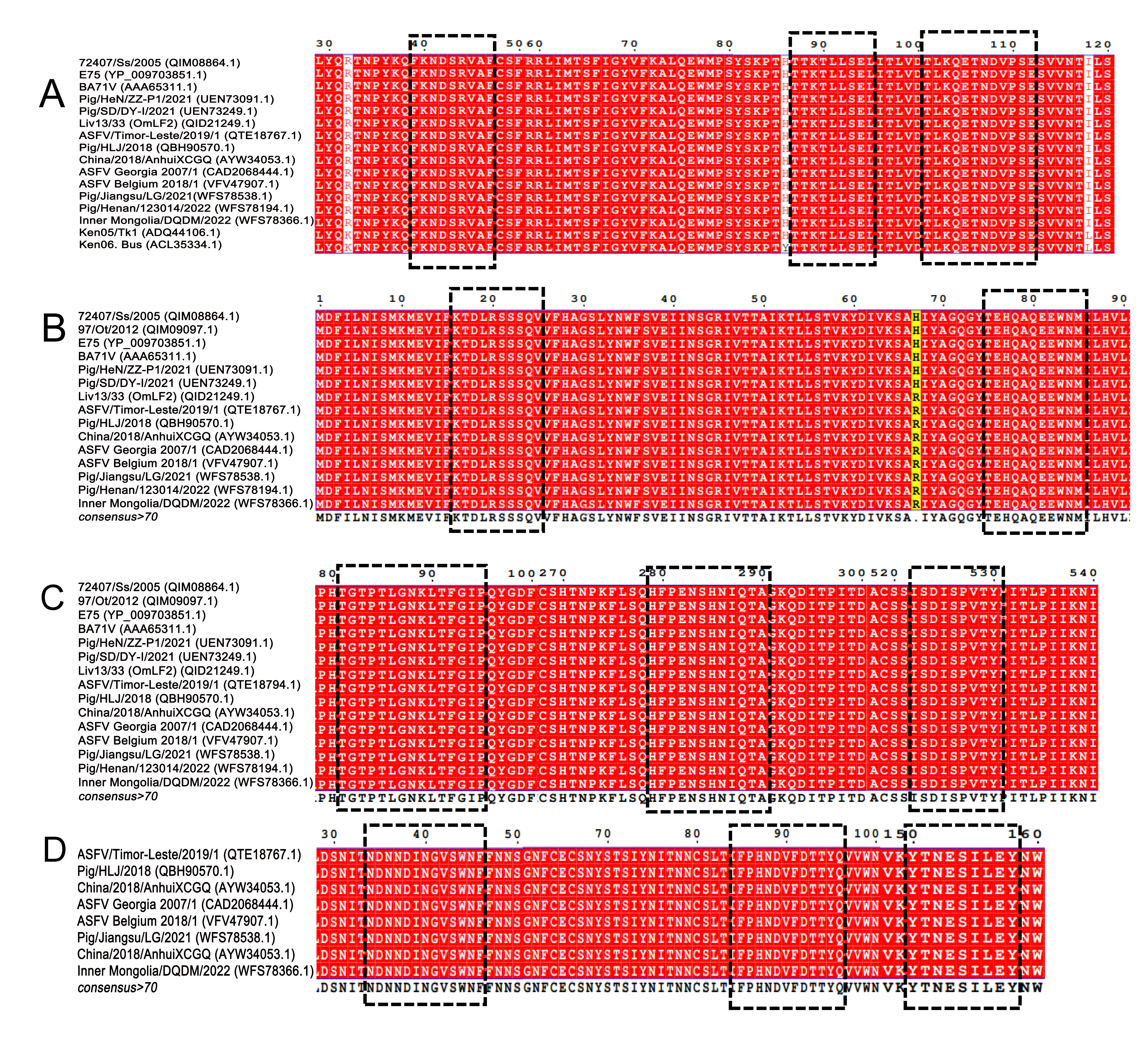

Supplement: Supplementary file 3 — Additional file 3: Figure S3. Epitopes are conserved in several pandemic strains of ASFV. A-D are sequence conservation analyses of ASFV pB602L, p30, p72, and CD2v, respectively. Identical colors indicate an exact match of amino acid residues. Homologous regions of identified protein epitopes are indicated by black dashed boxes. [file 12951_2023_2210_MOESM3_ESM.tiff]
